# Growth Hormone Receptor Mutations Related to Individual Dwarfism

**DOI:** 10.3390/ijms19051433

**Published:** 2018-05-10

**Authors:** Shudai Lin, Congjun Li, Charles Li, Xiquan Zhang

**Affiliations:** 1 Department of Animal Genetics, Breeding and Reproduction, College of Animal Science, South China Agricultural University, Guangzhou 510642, Guangdong, China; shudailin@stu.scau.edu.cn; 2 Guangdong Provincial Key Lab of Agro-Animal Genomics and Molecular Breeding, South China Agricultural University, Guangzhou 510642, Guangdong, China; 3 Key Lab of Chicken Genetics, Breeding and Reproduction, Ministry of Agriculture, South China Agricultural University, Guangzhou 510642, Guangdong, China; 4 Animal Genomics and Improvement Laboratory, Agricultural Research Service, United States Department of Agriculture, Beltsville, MD 20705, USA; congjun.li@ars.usda.gov; 5 Animal Biosciences and Biotechnology Laboratory, Agricultural Research Service, United States Department of Agriculture, Beltsville, MD 20705, USA; charles.li@ars.usda.gov

**Keywords:** growth hormone receptor, mutation, dwarfism, dysfunction

## Abstract

Growth hormone (GH) promotes body growth by binding with two GH receptors (GHRs) at the cell surface. GHRs interact with Janus kinase, signal transducers, and transcription activators to stimulate metabolic effects and insulin-like growth factor (IGF) synthesis. However, process dysfunctions in the GH–GHR–IGF-1 axis cause animal dwarfism. If, during the GH process, GHR is not successfully recognized and/or bound, or GHR fails to transmit the GH signal to IGF-1, the GH dysfunction occurs. The goal of this review was to focus on the *GHR* mutations that lead to failures in the GH–GHR–IGF-1 signal transaction process in the dwarf phenotype. Until now, more than 90 *GHR* mutations relevant to human short stature (Laron syndrome and idiopathic short stature), including deletions, missense, nonsense, frameshift, and splice site mutations, and four *GHR* defects associated with chicken dwarfism, have been described. Among the 93 identified mutations of human *GHR*, 68 occur extracellularly, 13 occur in *GHR* introns, 10 occur intracellularly, and two occur in the transmembrane. These mutations interfere with the interaction between GH and GHRs, GHR dimerization, downstream signaling, and the expression of *GHR*. These mutations cause aberrant functioning in the GH-GHR-IGF-1 axis, resulting in defects in the number and diameter of muscle fibers as well as bone development.

## 1. Introduction

Growth hormone (GH), also known as somatotropin, is a peptide hormone released from the anterior pituitary somatotroph cells. GH is involved in the promotion of growth, cell division and regeneration [1,2], the regulation of metabolism, immune, reproductive, and cardiovascular systems, and the brain [3,4]. GH effects are directly mediated through the GH receptor (GHR) and indirectly via insulin-like growth factor 1 (IGF-1). The secretion of GH is pulsatile, occurring under a variety of hormonal influences, such as stimulatory hypothalamic GH-releasing hormone, ghrelin and sex steroids, inhibitory somatostatin, IGF-1, and glucocorticoids. A complex feedback system involving IGF-1, leptin, ghrelin, free fatty acids, and the central nervous system regulates GH secretion. When released, GH binds to GHR in the liver and cartilage, leading to the production of IGF-1, which, through endocrine and paracrine/autocrine mechanisms, stimulates linear bone growth [5] or initiates other functions [6].

Notably, GHR plays a key role in the function of the GH–GHR–IGF-1 axis and is also an important factor for individual growth. In this axis, as an essential cytokine, GHR introduces the GH signal into the cell and then regulates the expression of IGFs, thereby regulating individual growth. Hence, the expression level and normal functioning of GHR in cells and tissues directly affects the physiological effects of GH [7,8]. Individuals with dysfunctional GHR, experiencing a loss or an abnormality in the GHR response to GH, are extremely short. They also have decreased bone mineral density and increased adiposity, with a greater risk of osteoporosis, lipid disorders, and cardiovascular disease [9].

Dwarfism is characterized by normal or elevated serum GH and low levels of IGF-1 [10]. Dwarf phenotypes exist in humans [3,7,11,12,13], mice [14], pigs [15,16], cattle, and sheep [17], and sex-linked dwarfism (SLD) occurs in chicken [2,18,19,20]. For example, Laron syndrome (LS), also known as growth hormone insensitivity syndrome, and idiopathic short stature (ISS) are autosomal recessive genetic disorders associated with severe postnatal growth failure and is mostly caused by mutation in the human *GHR*. Various mutations in the *GHR* gene have been reported, including deletion, RNA processing defects, translation stop codons, and missense mutations, which affect ligand binding, GHR dimerization, or signal transduction, which result in the failure to promote body growth [19,21,22]. A similar example is SLD in chicken, which is caused by *GHR* gene mutation, including point mutation in a structural gene or regulatory region, splicing site alterations, read frame shift, and complete or partial gene deletion. Moreover, SLD in chickens caused by different mutation types has a wide range of effects on production performance [18,19].

## 2. GHR Function and Process

### 2.1. GHR Structure

Human *GHR* cDNA encodes 638 amino acids, including the signal sequences of 18 amino acids (1–18 nt), an extracellular domain (ECD) of 246 residues (encoded by exons 2–7 of *GHR*), a transmembrane domain (IMD) of 24 residues (encoded by exon 8), and an intracellular domain (ICD) of 350 residues (encoded by exons 9 and 10). The ECD of the GHR includes two functional subdomains: subdomain 1 (residues 19–141) is involved in GH binding, and subdomain 2 (residues 146–264) is involved in receptor dimerization and GH-induced receptor rotation [23].

**Figure 1:**
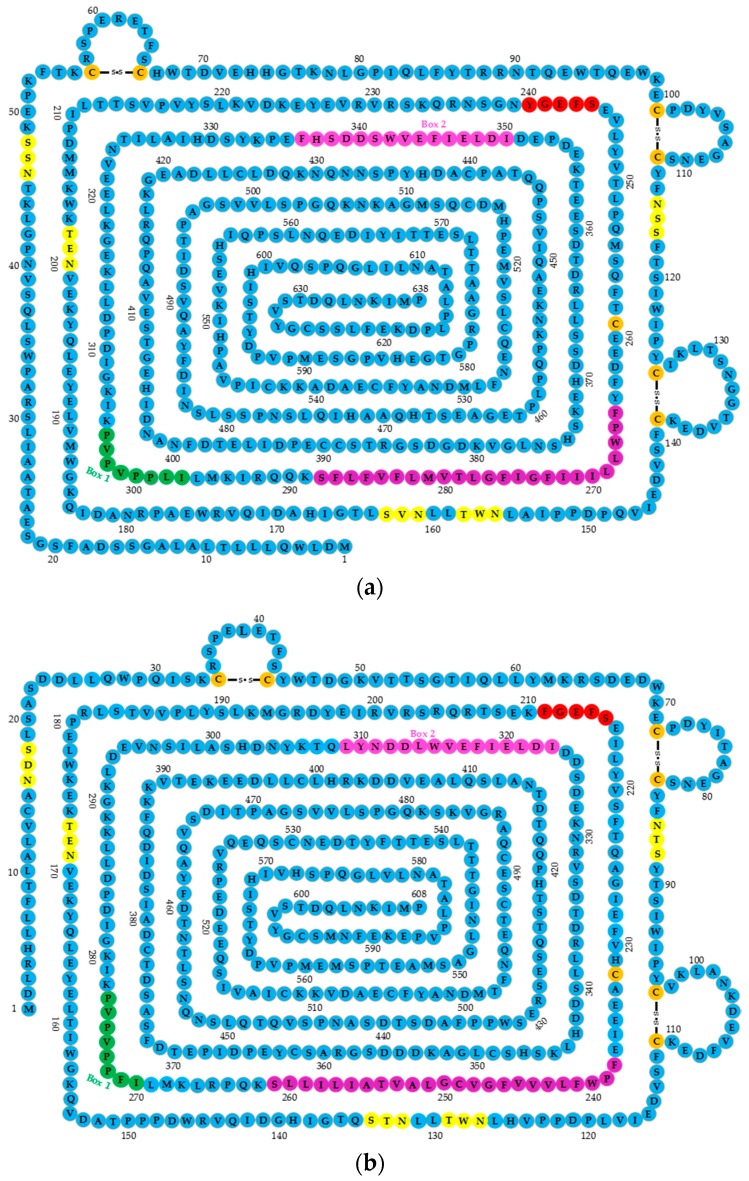
The structures of human and chicken growth hormone receptor genes (*GHRs*). (**a**) The structure of human *GHR*; (**b**) the structure of chicken *GHR*. The extracellular domain (ECD) contains five conserved glycosylation sites (yellow) (NXS/T, where X represents any amino acid), 7.5 cystine residues (orange), six of which are linked by disulfide bonds, and a WSXWS motif (240–244, shown in red); a transmembrane domain (TMD) shown in purple; and an intracellular domain (ICD), where Box 1 is shown in dark green, and Box 2 in pink.

The ECD contains five conserved glycosylation sites as a hormone binding domain; seven cysteine residues, of which six are linked by disulfide bonds, and the one at position 241 proximal to the membrane is free in the specific location that maintains the specific GHR extracellular segment spatial structure function; and a conserved binding domain near the cell membrane WSXWS motif. The human GHR motif is YXXFS, where X represents any amino acid. The motif is involved in changing the conformation of the binding of GH to GHR [24] (Figure 1a). In GHR ICD, the proline-rich Box 1 motif is located close to the cell membrane encoding eight amino acid residues, and the Box 2 sequence that encodes 15 amino acid residues includes aromatic and acidic residues (Figure 1a). These two conserved sequences (Boxes 1 and 2) play important roles in mediated signal transduction, and several tyrosine phosphorylation sites of GHR C-terminal domain represent many binding sites [3]. Compared with human *GHR* cDNA, that of chickens’ encodes 608 amino acids and lacks 20 amino acids (coded by exon 3) (Figure 1b). Many important motifs of chicken GHR are identical to human’s, such as the WSXWS motif in ECD, or Boxes 1 and 2 in ICD.

### 2.2. GHR Signal Transduction Mechanism

GH binds dimerized GHRs to form a trimolecular complex and then induces downstream signaling pathways [25]. The signal transduction mechanism of GHR is normally a four-step process: (1) one GH molecule and two GHR molecules recognize each other; (2) GH binds to two GHRs; (3) GH induces GHR_2_ dimerization forming a GH(GHR)_2_ complex; and (4) the GH(GHR)_2_ complex triggers signaling, such as Janus kinase 2, signal transducer and activator of transcription 5 (JAK2/STAT5), and Src family kinases [3,25,26,27].

#### 2.2.1. GH and GHR Recognition Event

Three distinct molecular recognition events are involved in GHR activation: two recognition events occur between GH of its two receptors separately, and the recognition that occurs between these receptors. The first two events involve ligand recognition, whereas the third involves the inter-recognition between two receptors that is essential for binding [3,28,29]. Molecule and ligand recognition involve different residues in both GH and the receptor interface that link together to form a GH–GHR complex [30]. Two distinct sites of GH, site 1 and site 2, must separately recognize GHR1 and GHR2 on the GHR dimer [3,26,28,30] to prepare for the next binding step that forms the GH(GHR)_2_ complex.

#### 2.2.2. GHR Dimerization

Different from the dimerization of other class 1 cytokine receptors induced by the cognate ligand, the two GHR molecules exist as a dimer at the cell surface before binding GH [3]. This suggests that the ECD is a place where the receptor–receptor interaction surface (site 3, involving the lower Fibronectin III domain) and the TMD are responsible for the dimerization of unliganded GHR [3]. Strikingly, subdomain 2 of GHR that includes six β-sheet regions, encompassing residues 135–263 (exons 6 and 7), is involved in receptor dimerization and GH induced receptor rotation [23,31]. This subdomain was found to determine the GHR dimerization partner [32].

#### 2.2.3. GH Binding to GHR Dimer

The binding of GH to the GHR dimer involves high-affinity binding [29] and occurs in the ECD subdomain 1 of GHR, composed of 19–142 (exons 2–5) [23,31]. The ECD of GHR includes a central hydrophobic patch dominated by Trp104 and Trp169, which are the central interaction sites for GH [33]. The GH molecule has two sites: site 1, which is a high-affinity binding site, and site 2, a low-affinity binding site, each of which interacts with the ECD of the preformed GHR dimer. GH site 1 first interacts with one of the GHRs and then site 2 interacts with the other GHR to create a functionally or properly dimerized complex that ultimately induces intracellular signaling. The interaction of GH with the GHR dimer causes the rotation of the two GHRs relative to each other [34].

This means the combination of a GH molecule with two GHR molecules requires two steps: rotation and redirection of the two GHR molecules. The combination leads to the activation of the related kinase and cross-phosphorylation. Each GHR cytoplasm tail phosphorylates, calls up and then phosphorylates the cytoplasmic protein, triggering further phosphorylation cascade reactions and transcriptional activation [3,34].

#### 2.2.4. GHR Mediating GH to Janus Activating Tyrosine Kinase (JAK)/STAT Signature Transduction

GH binding of the GHR dimer promotes rotation and changes the conformation of the GHRs, ultimately activating JAK2 to initiate the intracellular signaling cascade [3]. As mentioned above, Boxes 1 and 2 in the ICD of GHR play important roles in GH–GHR–IGF-1 axis signal transduction. These two domains are JAK2 binding motifs, which were coupled with and activated by JAK2. The JAK2 binding motifs in the GHR dimer are responsible for controlling the position of the GHR transmembrane helices, atomistic modeling of the transmembrane helix movements, and docking the crystal structures of the JAK2 kinase. After the tyrosine phosphorylation of the JAK2 molecules, several GHR intracellular tyrosines are phosphorylated, which provides docking sites or enables the binding of adaptor proteins such as STAT5 [35].

JAK phosphorylates not only certain tyrosine residues in the receptor cytoplasmic domain, but also other protein substrates directly. Key tyrosine residues in the GHR cytoplasmic domain serve as docking sites for STAT5 and other SH2-domain-containing proteins, which mediate a considerable part of GH action at the genome [36]. Docking of these transcription factors facilitates their phosphorylation by JAK2, subsequent STAT5 dimer formation, and translocation to the nucleus where the dimer regulates the target gene [3,35].

## 3. GHR Polymorphisms and Individual Dwarfism

GH affects growth by binding to GHR in target cells. Given the biological effects exerted by GH through GHR, deletions and mutations in the *GHR* gene may affect the expression or function of GHR, after disrupting the effects of the GH/IGF-1 pathway. Consequently, compared to the average individual height and weight in a population, skeletal development is different. Individual dwarfism caused by GHR disorders are LS and ISS in humans [12,13,22,37,38,39], miniature pigs [15,16,40], cattle and sheep [17], and sex-linked dwarfism (SLD) in chickens [2,18,20].

GHR function abnormalities are primarily related to mutations of the gene structure, including base mutation and fragment deletion. Since the late 1990s, researchers discovered many *GHR* mutations, including deletion, nonsense, missense, frameshift, splicing site, and large fragment deletion mutations [38,41]. The variations in *GHR* gene structure affect the structure and function of its expressed protein, so that GH is unable to bind to the hepatocyte membrane, resulting in growth inhibition, showing a dwarf phenotype.

### 3.1. GHR Gene Mutations that Causing Aberrant GH–GHR Binding

Mutations in the *GHR* gene can alter the ability of *GHR* to interact with the GH peptide [8]. Nearly 30 *GHR* mutations have been reported to be associated with disturbing the GH–GHR combination (Table 1 and Table 2). For example, a homozygous substitution mutation E42K was predicted to impair the binding affinity of GHR to GH in humans, and to be responsible for low serum levels of IGF-1, IGF binding protein (IGFBP)-3, and GH binding protein (GHBP) [42]. A nonsense mutation in the fourth exon of *GHR* (R43X) determines a premature termination in the protein translation process. As a result of the absence of the extracellular portion of the GHR, this patient had undetectable GHBP [43]. GHR C94S was found to lose its ability to bind to GH [44]. A 307 G > A substitution in exon 5 of *GHR* resulted in the replacement of the amino acid aspartic acid at position 103 with a residue of asparagine (D103N). This substitution involved the highly conserved aspartic acid 103, which could be responsible for damaging GHR functionality [45]. Mutations identified in humans with short stature, localized in the ECD of *GHR*, are responsible for impairing GH binding. Although treating patients with high doses of recombinant human GH (rhGH), produced some IGF-1 for a short time, due to the failure of the compensatory mechanisms, the IGF-1 was insufficient for normal growth, delaying bone age, consequently affecting final height [45,46].

For chickens, altering one base (T335C) in *GHR* exon 5 resulted in an amino acid changes (F112S) in SLD chicken. Since this site is located proximal to a disulfide bond and is a GH binding site, this substitution reduces GH binding activity on the hepatocyte membrane to less than 10%. This base mutation may cause GH failure or impair the ability of GH to bind to the GHR, reducing or stopping the GH synthesis and secretion of IGF-1 through the GH–GHR–IGF-1 axis, ultimately leading to the growth inhibition phenomenon [11]. S226I missense mutation in exon 7 was found to be related to SLD chickens because of the deletion of large fragments [47] (Table 3).

### 3.2. GHR Gene Mutations Causing Aberrant GHR Dimerization

Mutations occurring in the dimerization domain (exons 6–8) of GHR affect the formation of the GHR dimer. For instance, heterozygous R179C non-synonymous mutation occurs in *GHR* exon 6 [48], and two other mutations as well. The first is E180X (GAA > TAA), which activates a cryptic splice acceptor resulting in a receptor protein with an 8-amino acid deletion in the extracellular dimerization domain. Although retaining the ability to homodimerize, trafficking to the cell surface was defective [39]. The second is E180 splice, which affects both GH binding and GHR trafficking, rendering the abnormal GHR nonfunctional [38].

Furthermore, a deletion of 166 bases of exon 7 resulted in premature termination (M207 fs. X8). This mutation decreases GH binding affinity to the GHR, and would thus be responsible for growth retardation [49] (Table 2).

### 3.3. GHR Mutations Causing GHR Failure of GH Delivery to Downstream Genes

**Figure 2:**
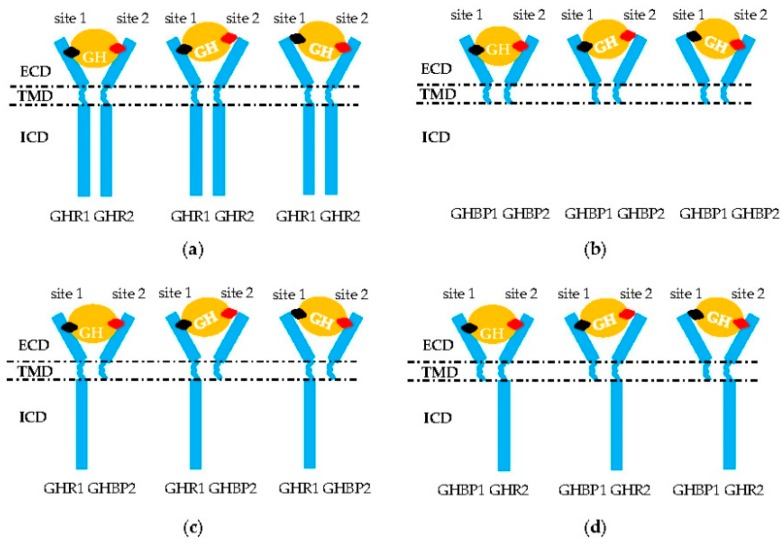
Aberrant signal transduction of GH into the intracellular domain (ICD) of GHR. (**a**,**b**) Homodimerization of two GHR or GHBP molecules. (**c**,**d**) Heterodimerization of one GHR and one GHBP molecule or/and GH failing to combine in the GHR–GHBP dimer. Only GH (orange circle) site 1 (black rhombus) or 2 (red rhombus) bind successfully to one molecule in these two homodimers that leads to a failure in the formation of the GH–GHR_2_ complex. ECD: extracellular domain; TMD: transmembrane domain; ICD: intracellular domain of GHR.

The GHR_2_ dimers must be combined by GH sites 1 and 2 before performing its transduction function (Figure 2a) [3]. Curiously, mutations localized in the whole sequence of the *GHR* gene are associated with the defective pathogenesis of the signal transmission. For example, the affinity of GHR to GH remains normal with GHR H150Q, but signal transduction capacity is inhibited (Table 1) [44]. For chicken, the 1.7 kb deletion in the 10 and 3’UTR exon regions is responsible for dwarf chickens [18]. The mechanism of this mutation is that the lost loci identified by microRNA let-7b can suppress the expression of the *GHR* gene. Then, an excess of GHR occurs, which leads to adipose deposition and repressed growth [2]. Mutations at the cleavage sites resulted in an inability of the transcripts to cleave normally (Table 3) [11]. Affecting the critical JAK2-binding Box 1 region of GHR ICD (p.R229H/c.899dupC) can lead to a frameshift and early protein termination, disrupting normal GHR signaling [23,63]. In addition, deleting the proline-rich region, or changing the four prolines to alanines, also resulted in GHR deficient signaling [64]. Furthermore, alternative splicing of the *GHR* precursor, mRNA, and truncation of GHR can lead to the synthesis of signaling incompetent GHR. For example, deleting exon 3 GHR represented by a 532-bp fragment terminated the signal transmission in advance [37,51]. Truncated GHR protein resulting from exon 8 skipping was directly secreted out of the cell [65], and truncated GHR missing 184 amino acids and another truncated GHR lacking all but five amino acids of the cytoplasmic domain could not mediate any effects of GH, nor was it internalized [64]. Truncated GHR 1–277 and 1–279 variants led to a translational frame shift that introduced a stop codon three to four amino acids after the GHR TMD, leading to truncation of the entire cytoplasmic domain (Table 4) [6,66]. Using rhGH or rhIGF-1 to treat the patients with these mutations, although serum IGFBP-3 was normalized or below normal, IGF-1 serum levels were only modestly increased. This means that patients would either lack a response to rhGH or rhIGF-1, which would inhibit downstream GH-induced signaling through the negative feedback loop to the pituitary [63].

Two truncated GHRs formed a heterodimer (Figure 2b), but further study is required. Sometimes these truncated GHR produced GHBP [30]. Reports suggest a predominantly negative effect of truncated variants, as they can form a long–short heterodimer with a full-length GHR, which hampers the dimer signaling (Figure 2c,d) [67]. Considering GHR is conserved among different species [68,69], this kind of phenomenon can occur with other mammal and avian species.

### 3.4. Mutations Resulting in GHR Expression Failure

A missense mutation caused by the transformation of adenine to guanine (c.1 A > G) was found in the first codon of exon 2. Given that this substitution involved the translation initiation codon of the protein, the correct expression of the receptor is inhibited [45]. The sequencing for *GHR* exon 5 revealed a TT insertion at nucleotide 422 after codon 122. The insertion resulted in a frameshift introducing a premature termination codon that led to a truncated receptor (Table 1) [22]. Additionally, a splice site mutation was located at the donor splice site of exon 2/intron 2 within *GHR*, changing the open reading frame of *GHR*, resulting in a premature termination codon in exon 3 (Table 5) [10]. Also, a subset of the GHR homodimer was cleaved at the cell membrane, releasing the ECD known as the GHBP into the circulation. The mechanism underlying the generation of soluble GHBP likely differs between species. Human *GHR* truncation is identical in sequence to full-length *GHR*, except for a 26-bp deletion, leading to a stop codon at position 280, thereby truncating 97.5% of the intracellular domain of the receptor protein. When compared with human *GHR*, human truncated *GHR* showed a significantly increased capacity to generate soluble GHBP [70]. Another example is the G679T substitution at the *GHR* gene leading to the replacement of S226I. Although the length of the encoded protein did not change, this protein was not expressed on the surface of hepatocytes [60]. The GHBP is a transcriptional activator in mammalian cells, and this activity occurs in the lower cytokine receptor module. This activity is dependent on S226, the conserved serine of the cytokine receptor consensus WSXWS box [1]. A homozygous 784 G > C transversion induced exon 7 excision and the functional loss of the native intron 7 donor splice site, leading to a frame shift and predicted early protein termination [62].

Patients with growth hormone insensitivity and without mutations in the *GHR* gene coding region should be screened for mutations in the noncoding regions, such as an intronic *GHR* mutation within intron 4 (266 + 83 G > T), which generates a 5’ donor splice site to retain 81 intronic nucleotides in the *GHR* mRNA. The abnormal splicing event caused early protein termination and undetectable GHBP in the serum [66]. Intron 6 (A (−1) > G (−1)) substitution lead to the skipping of exon 6 and premature termination of the mRNA message (Table 5) [12,44]. All the mutations in *GHR* introns are splice site mutations, which disrupt the expression of GHR.

### 3.5. GHR Regulates Development of Both Bone and Muscle Fiber

In addition to promoting linear growth, GH plays a crucial role in the regulation of bone and muscle development and metabolism by acting directly through the GHR [73,74], or indirectly via hepatic IGF-1 production. Signal transduction errors in the GH–GHR–IGF-1 axis can cause growth failure and changes in body composition. GHR signaling in bone is necessary for establishing radial bone growth and optimizing mineral acquisition during growth [75].

Longitudinal growth is primarily influenced by the GH–IGF-1 axis, which is a mixed endocrine–paracrine–autocrine system [76]. Constant manipulation of the GH–IGF-1 axis influences both morphology and mRNA levels of selected genes in the muscle–tendon units of mice. However, only moderate structural changes were observed with up-regulation of the GH–IGF-1 axis; disrupting of the GHR had pronounced effects upon tendon ultrastructure [77]. GH directly impacts the growth plate to stimulate longitudinal growth, demonstrated by staining the GHR and GHBP located in both the cytoplasm and the nucleus. The localization of GHR/GHBP suggests that, in addition acting on germinal and proliferative cells in young rats, GH also affects early-maturing chondrocytes and may be involved in their differentiation to a fully hypertrophic chondrocyte [78]. Mutation of the *GHR* gene also caused a decrease in the number of muscle fibers, decreasing the myofiber diameter [79]. GHR signaling in postnatal skeletal muscle was not found to play a significant role in regulating muscle mass or muscle regeneration [80].

## 4. Discussion

It is well known that there are two different steps of GH action: at first, GH is directly mediated by GHR to be transducted to IGF1; and then the IGF-1 acts on target cells to exert the physiological effect of GH [4,81]. Although GH acts in nearly every tissue of the body, the most known for its growth promoting effect of GH is on cartilage and bone, especially during the adolescent years. Once the JAKs/STATs signal was activated by GH, they were transported into the nucleus to induce increased gene transcription and metabolism to produce IGF-1, which will release into the circulatory system. IGF-1 then binds to its receptor on the cellular surface and activates a JAK-mediated intracellular signaling pathway, which intracellularly phosphorylates various proteins leading to increased metabolism, anabolism, and cellular replication and division [82].

The effects of GH were mediated by systemic IGF-1, so it should be assumed as a combined effect of both GH and IGF-1. Pituitary GH is the major regulator of liver-produced IGF-I, which is transported via the circulation to peripheral tissues where it acts in an endocrine manner [83]. However, several evidences support the notion that circulating IGF1 is independent of GH. Glucose, leptin, insulin and proluctin can stimulate the expression of IGF-1 in patients without GH to maintain their normal growth [84]. Moreover, it was well demonstrated that IGFs regulate bone length of the appendicular skeleton evidenced by changes in chondrocytes of the proliferative and hypertrophic zones of the growth plate. IGFs affect radial bone growth and regulate cortical and trabecular bone properties via their effects on osteoblast, osteocyte, and osteoclast function [83].

Notably, the functionality of the GH–GHR–IGF-1 axis partially depends on the expression of liver GHR, which determines the amount of IGF1 released from the liver in response to GH [81]. The loss of GHR leads to a decrease in the synthesis and secretion of IGF-1 [85]. The net effect of the loss in IGF1 is that leads to a loss of negative feedback on GH, as well as cells may be systemically starved for growth factors and have slower growth or less metabolic activity [81].

We summarized different mutations of the *GHR* gene and their possible mechanism relevant to human or chicken’s dwarfism. *GHR* mutations that occur in the encoding region may cause amino acid changes, thus affecting protein structure and function. Mutations of the splicing sites result in the improper translation of transcripts into biologically active proteins. Deletion mutations lead to a frameshift, affecting the structure and function of proteins. Even in regulatory regions, mutations may affect the temporal and spatial expression patterns of the gene.

Several defects have been reported to be associated with body dwarfism. For instance, the G62V mutation in exon 4 [53], a heterozygous mutation (V144I) within exon 6 of the *GHR* [13], a dinucleotide deletion on exon 7 of the *GHR* gene [61], and even a GT-repeat microsatellite in the *GHR* 5’UTR [7] can result in dwarfism. However, not all mutations in *GHR* cause body dwarfism. For example, mutation of phenylalanine 346 to alanine resulted in a GHR that did not internalize rapidly. However, this mutant GHR was capable of mediating GH-stimulated transcription as well as having metabolic effects [64]. S325S, L526I [86], c.–10 T > C (exon 2), G168 (exon 6) and 8 intronic mutations of GHR (662 − 31 C > T, 662 − 30 A > G, 662 − 24delG, 662 − 11delT, 482 + 9 C > T, 828 − 4delG, 919 − 14delT, and 988 + 23delG) [87] did not induce individual dwarfism. However, the dose individually affects the *GHR* mutations causing dwarfism. Various mutations in *GHR* genes occur in some individuals, producing additive effects that lead to individual dwarf performance [87]. For example, the dwarf (*dw*) gene in chickens creates a loss of function mutation with a dose effect because the offspring of SLD cocks and normal hens only showed a slight decrease in body weight. From the point of view of traditional genetics, multiple alleles in the dw locus, and the genetic effects of these alleles are also different. When multiple alleles exist at the same time, they may also have additive effects. Similar findings were also found in the pig melanin receptor gene [88].

Normally, after the GHR–GHR homodimer binding to GH, each GHR molecule is coupled with one or more non-activated kinases through a non-covalent form, including JAK2 and Src family kinases [34]. However, except for GHR–GHR homodimerization, GHBP–GHBP homodimerization and GHBP–GHR heterodimerization [63,89] reactions are pervasive mechanisms that hinder signal transduction [28]. Diagnosis and treatment of these abnormal phenomena are formidable tasks for researchers.

A transcription of the *GHR* gene (GHRG-1, coding 120 amino acids) [90] and an antisense transcript of *GHR* (4337 bp, non-coding RNA) [91] were only expressed in normal chickens and are considered as new GH regulators. Researchers demonstrated that the dwarf phenotype of the SLD chicken is not only controlled by the full-length *GHR*, but it may also be regulated by different transcripts of *GHR*. Given the conservativeness of *GHR* gene expression in humans and chickens, this process may also contribute to human dwarfism.

Mutations in *GH* and *IGF-1* genes are also important when studying body dwarfism, because the mutations of both genes may play roles altering the GH–GHR–IGF-1 axis signaling transduction. GH has no axis of symmetry. Its interaction with GHR is mediated by two distinct asymmetric binding sites with different affinities on GH. Site 1 has high affinity and mediates the first binding step. Mutation of binding site 2, as with the human GH mutant G120R, disrupts the second binding but leaves site 1 binding intact. G120R is a GH antagonist, binding only one GHR and thus fails to signal, and it prevents productive GHR binding by normal GH [25]. The mechanism by which GH binding converts the inactive pre-dimerized GHR to its active signaling conformation has not been confirmed.

## 5. Conclusions

To date, 93 *GHR* mutations related to human dwarfism and 4 *GHR* mutations associated with chicken dwarfism have been described. This study contributes to our understanding of *GHR* mutations, including fragment deletions, point, missense, nonsense, splice site, and frameshift mutations, which not only contribute to individual dwarfism but also influence body bone development. Dwarfism in an individual may be caused by more than one kind of *GHR* mutation and may result from defects in others genes.

## Figures and Tables

**Table 1:** Mutations in the extracellular domain (ECD) of *GHR* relevant to human dwarf mainly by interfering with GH–GHR binding.

Location	Base Mutation	Defect	Mutation Type	Mechanism	References
Exon 2	c.1 A > G ^1^	M18V	Missense	Inhibits the correct expression of *GHR*	[45]
	c.1 A > T	M18L	Missense		[50]
	c.12 G > A	W15X	Nonsense	Introduces a premature termination codon that leads to a truncated non-functioning receptor	
Exon 3	c.101 G > A	W16X	Nonsense		
	71–136 del ^2^	7–22 del	Deletion 7–22		[37]
	del	exon 3del	Frameshift	d3-GHR, represented by a 532-bp fragment, forms a nonfunctional receptor that terminates the signal transmission in advance	[37,51]
Exon 4	c.162 delC	36delC	Frameshift	May interfere with GH binding activity	[41]
	c.166 T > A	C38S	Missense	May affect GH binding activity	[52]
	c.168 C > A	C38X	Nonsense		
	c.173 C > T	S40L	Missense	May interfere with GH binding activity	[10]
	c.180 G > A	E42K	Missense	Impairs the GHR binding affinity to GH	[42]
	c.181 C > T	R43X	Nonsense	Causes undetectable GH binding protein (GHBP)	[43]
	c.192_193delTT	46delTT	Frameshift		[52]
	c.202 T > C	W50R	Missense	May affect the GH binding activity	[41]
		G62V		Be associated with idiopathic short stature	[53]
	c.247 C > T	Q65X	Nonsense	Could interfere with GH binding activity	[41]
	c.249 T > C	S65H		Lowers serum levels of IGF-1, IGFBP-3, and GHBP compared to normal controls	[54]
	c.266 G > A	R71K	Missense		[52]
	del(5) p11–p13.1	del exon 4–10	Nonsense		[50]
Exon 5	c.293 G > A	W80X	Nonsense	Could interfere with GH binding activity	[41]
	c.303 C > A	C83X	Nonsense	Leads to a lack of GHR expression as a result of mRNA decay or defect in cell membrane anchoring	[55]
	c.310 T > G	Y86D	Missense	Located in one of the two cysteine-rich regions of GHR	[50]
	c.335 G > C	C94S	Missense	Loses the ability of binding to GH	[44]
	c.337 T > C	C94C	Synonymous	Delays bone age	[46]
	c.341 T > C	F96S	Missense	Interferes with GHR intracellular transport to the cell membrane	[52]
	c.338dupA ^3^	Y97X	Nonsense	Causes truncated GHR and a loss in receptor function because it lacks amino acids comprising the transmembrane and intracellular regions of the GHR protein	[50]
	307 G > A	D103N		Responsible for impaired GH binding that alters receptor functionality	[45]
		W104R			[37]
	c.421_422dupTT	422insTT	Frameshift	Results in a frameshift that introduces a premature termination codon that leads to a truncated receptor	[22]
	c.428 T > C	V125A	Missense		[10]
	~1.2 kb deletion		Deletion	Skipping the truncated exon 5 leads to a frameshift and a premature termination codon	[50]
	~4 kb deletion		Deletion	Leads to a stop codon in exon 6 that predicts a truncated, non-functional GHR protein	
	del	exon 5del	Frameshift	Forms a nonfunctional receptor that terminates the signal transmission in advance	[56]
Exons 5 and 6	~19 kb deletion		Deletion	Deletion of a large portion of the ECD, hormone binding domain of GHR	[50]

^1^ c.: coding sequence; ^2^ del: deletion; ^3^ dup: duplicate.

**Table 2:** Mutations in the *GHR* dimerization domain relevant to human dwarfism.

Location	Base Mutation	Defect	Mutation Type	Mechanism	References
Exon 6		P131Q	Missense		[52]
	c.476 T > A ^1^	L141X	Nonsense	Introduces a premature termination codon that leads to a truncated non-functioning receptor	[50]
	c.484 G > A	V144I		Associated with idiopathic short stature	[13]
	c.484 G > T	V144F			[46]
	c.485 T > A	V144D	Missense		[52]
	c.485 T > C	V144A			[46]
	c.504 T > G	H150Q	Missense		[44]
	c.508 G > C	D152H	Missense	Disrupts the expression, dimerization, and signaling of GHR	[52]
		D152G	Missense		[57]
	c.512 T > C	I153T	Missense	Mainly affects intracellular trafficking and binding affinity of the receptor	[50,58]
	c.515 A > C	Q154P	Missense	Leads to severe defects both at the cell surface and in total particulate membrane fractions	
	c.518 T > G	V155G	Missense	Affects intracellular trafficking and binding affinity of GHR	
	c.524 G > A	W157X	Nonsense	Produces a truncated GHR, lacking part of exons 6 and 7–10, defective in both cell membrane anchoring and GH binding	[41]
	c.535 C > T	R161C	Missense	Causes low serum GHBP concentrations	[52]
	c.558 A > G	G168G		Deletes the ECD and forms a nonfunctional receptor that terminates the signal transmission in advance	[10]
	c.559 T > C	W169R		Since Trp169 plays an important role in the stabilization of the GH–GHR interaction, this mutation in chain 1, which binds to GH site 1, showed a decreased affinity for GH, affecting the interaction in the complex	[8]
	c.591 C > T	R179C			[48]
	c.594 G > T	E180X	Nonsense	Deletes the ECD and forms a nonfunctional receptor, which terminates the signal transmission in advance	[21,44]
	c.594 A > G	E180sp ^2^	Splice site	Causes deletion of residues 181–188 in the dimerization functional region	[44]
	c.601 G > T	E183X	Nonsense		[50]
	del ^3^	deletion of exon 6	Frameshift	Results in a deletion of a large portion of the ECD of GHR	[50]
Exon 7		M207 fs. X8	Deletion	Results in premature termination, which decreases GH binding affinity	[49]
	c.677 A > G	Y208C	Missense	Prevents normal interactions in the membrane proximal domain of the extracellular part of the receptor	[50]
	c.685 C > G	R211G	Missense		[52]
	c.703 C > T	R217X	Nonsense	Deletes the ECD and forms a nonfunctional receptor, which terminates the signal transmission in advance	[21]
	c.723 C > T	G223G	Splice site	May interfere with GH binding activity	[41]
	c.724 G > T	G224X	Nonsense		[52]
	656 C > T	p.S219L ^4^			[59]
	c.731 G > T	S226I	Missense	Occurs in WSXWS-like motif of GHR causing GH insensitivity	[60]
		R229H			[23]
	a nucleotide del	del203AT or TA	Frameshift	Results in high GH levels and low levels of IGF-1, IGF-2, IGFBP 3, and GHBP	[61]
	c.743_744 del AT	230delAT	Nonsense		[52]
	c.766 C > T	G236sp	Splice site	Activates the cryptic splice donor site within exon 7	
	c.784 G > C	D244N	Missense	Induces functional loss of the native intron 7 donor splice site, leading to a frame shift and predicted early protein termination	[62]
Exon 8	c.875 G > C	R274T	Splice site	Generates a truncated protein	[52]
		S473S			[37]

^1^ c.: coding sequence; ^2^ sp: splice; ^3^ del: deletion; ^4^ p.: pre-peptide.

**Table 3:** Mutations in GHR relevant to sex-linked dwarfism (SLD) in chicken.

Location	Base Mutation	Defect	Mutation Type	Mechanism	References
Exon 5	c.335 T > C ^1^	F122S	Missense	Causes a large decrease in GH binding activity	[11]
	c.352+2 T > C		Splice site	Reduces GH binding activity	[11]
Exon 7	c. 679 G > T	S226I	Missense	Protein not expressed on the surface of hepatocytes	[10,47]
Exon 10 and 3’ UTR ^2^	c.1773 del ^3^		Frameshift	Lacks the target site for microRNA let-7b, which down-regulates the *GHR* expression level	[2,18]

^1^ c.: coding sequence; ^2^ UTR: untranslated region; ^3^ del: deletion.

**Table 4:** Intracellular domain (ICD) mutations of GHR relevant to human dwarfism by preventing GHR downstream signaling.

Location	Base Mutation	Defect	Mutation Type	Mechanism	References
Exon 9	removal of exon 9	truncated GHR 1–279	Frameshift	Removal of 26 base pairs of exon 9 does not have direct signaling function, but can form a long–short heterodimer with full-length GHR, inhibiting the STAT5 signal of full-length GHR and may therefore play a significant role in regulating the function of wild-type GHR	[6]
	c.889_911del ^1,2^	889_911del		Results in an intracellular trafficking of GHR	[50]
	c.899dupC ^3^			Influences the critical JAK2-binding Box 1 region of the GHR ICD; the duplication predicts early protein termination	[23]
	G920_921insTCTCAAAGATTACA	truncated		Robustly expressed as truncated, fails to activate STAT5B signaling	[63]
	c.945 + 2 T > C	lost Box 1		Excision of exon 9 that can form a long–short heterodimer	
Exon 10	c.964dupG	truncated		Robustly expressed as truncated, fails to activate STAT5B signaling	
	c.981delC	309delC		Causes the production of 20 novel amino acids (310–329) instead of the wild-type sequence, premature termination at codon 330, and the subsequent deletion of the C terminal portion of the intracellular domain	[52]
	c.1342_1345del	GHR (1–499)		Truncated after Box 1, which results in the isolated failure of STAT 5 signal transduction	[50]
	c.1734delG	1776delG		Lower STAT5-mediated transcriptional activation	[55]
		I544L		Deletes the extracellular domain and forms a nonfunctional receptor, which terminates the signal transmission in advance	[21]

^1^ c.: coding sequence; ^2^ del: deletion; ^3^ dup: duplicate.

**Table 5:** Mutations in introns of *GHR* relevant to human dwarfism due to the failure of *GHR* expression.

Location	Base Mutation	Defect	Mutation Type	Mechanism	References
Exon 2-intron 2	GT > GGT		Splice site	Results in an immature stop codon in exon 3	[10]
Intron 2	c.70 + 1 G > A ^1^	70 + 1 G > A		May interfere with GH binding activity	[37]
Intron 4	c.266 + 1 G > A	71 + 1 G > A		Destroys the splice donor and acceptor invariant sequences of consensus sites for mRNA processing	[52,71]
	c.266 + 83 G > T			Results in retention of 81 intronic nucleotides in the *GHR* mRNA that leads to early protein termination	[65]
Intron 5	c.440 − 1 G > C	IVS5 − 1 G > C		Destroys the splice donor and acceptor invariant sequences of consensus sites for mRNA processing	[71]
Intron 6	c.618 + 18kb A > G	ψ6		Leads to recognition of the pseudoexon and inclusion of an additional 108 bases between exons 6 and 7 that adds 36 amino acids in the GHR ECD	[72]
	c.619 + 1 G > A	IVS6 + 1 G > A		Leads to the skipping of exon 6 and premature termination of the mRNA	[12,44]
	c.619 − 1 G > T	189 − 1 G > T			[71]
	c.619 − 1 G > C	189 − 1 G > C			[50]
	c.619 − 25 A > G	IVS6 − 25 A > G			
Intron 7	c. 785 − 1 G > T	785 − 1 G > T		Results in a truncated protein that retains GH binding activity and is probably no longer anchored in the cell membrane, affecting signal transmission	
Intron 8	c.876 − 1 C > G	GHR (1–277)		Truncates the ICD of the GHR, which could form a heterodimer with the wild-type GHR, the activity of which is inhibited in a dominant-negative manner	[6,52]
Intron 9	c.945 + 1 G > A	GHR (1–277)		Produces a truncated protein with deletion of 98% of the ICD of the GHR, including Boxes 1 and 2, resulting in failure of GH signal transduction and GHR internalization	[52,67]

^1^ c.: coding sequence.

## Abbreviations

GH	Growth hormone
GHR	GH receptor
GHBP	GH binding protein
IGF	Insulin-like growth factor
LS	Laron syndrome
SLD	Sex-linked dwarf
ECD	Extracellular domain
TMD	Transmembrane domain
ECD	Intracellular domain
JAK	Janus kinase
STAT	Signal transducers and activators of transcription

## References

[1] Conway-Campbell B.L., Brooks A.J., Robinson P.J., Perani M., Waters M.J. (2008). The extracellular domain of the growth hormone receptor interacts with coactivator activator to promote cell proliferation. Mol. Endocrinol..

[2] Lin S., Li H., Mu H., Luo W., Li Y., Jia X., Wang S., Jia X., Nie Q., Li Y. (2012). Let-7b regulates the expression of the growth hormone receptor gene in deletion-type dwarf chickens. BMC Genom..

[3] Waters M.J. (2016). The growth hormone receptor. Growth Horm. IGF Res..

[4] Martínez-Moreno C., Calderón-Vallejo D., Harvey S., Arámburo C., Quintanar J. (2018). Growth Hormone (GH) and Gonadotropin-Releasing Hormone (GnRH) in the Central Nervous System: A Potential Neurological Combinatory Therapy?. Int. J. Mol. Sci..

[5] Reh C.S., Geffner M.E. (2010). Somatotropin in the treatment of growth hormone deficiency and Turner syndrome in pediatric patients: A review. Clin. Pharmacol..

[6] Soendergaard C., Young J., Kopchick J. (2017). Growth Hormone Resistance—Special Focus on Inflammatory Bowel Disease. Int. J. Mol. Sci..

[7] Dias C., Giordano M., Frechette R., Bellone S., Polychronakos C., Legault L., Deal C.L., Goodyer C.G. (2017). Genetic variations at the humangrowth hormone receptor (GHR) gene locus are associated with idiopathic short stature. J. Cell. Mol. Med..

[8] Porto W.F., Marques F.A., Pogue H.B., de Oliveira Cardoso M.T., Do Vale M.G.R., Da Silva Pires Á., Franco O.L., de Alencar S.A., Pogue R. (2017). Computational Investigation of Growth Hormone Receptor Trp169Arg Heterozygous Mutation in a Child With Short Stature. J. Cell. Biochem..

[9] Rosenbloom A.L. (2016). A half-century of studies of growth hormone insensitivity/Laron syndrome: A historical perspective. Growth Horm. IGF Res..

[10] Arman A., Ozon A., Isguven P.S., Coker A., Peker I., Yordam N. (2008). Novel splice site mutation in the growth hormone receptor gene in Turkish patients with Laron-type dwarfism. J. Pediatr. Endocrinol. Metab..

[11] Hull K.L., Marsh J.A., Harvey S. (1999). A missense mutation in the GHR gene of Cornell sex-linked dwarf chickens does not abolish serum GH binding. J. Endocrinol..

[12] Hui H.N., Metherell L.A., Ng K.L., Savage M.O., Camacho-Hubner C., Clark A.J. (2005). Novel growth hormone receptor mutation in a Chinese patient with Laron syndrome. J. Pediatr. Endocrinol. Metab..

[13] Pagani S., Petkovic V., Messini B., Meazza C., Bozzola E., Mullis P., Bozzola M. (2014). Heterozygous GHR gene mutation in a child with idiopathic short stature. J. Pediatr. Endocrinol. Metab..

[14] Sadagurski M., Landeryou T., Cady G., Kopchick J.J., List E.O., Berryman D.E., Bartke A., Miller R.A. (2015). Growth hormone modulates hypothalamic inflammation in long-lived pituitary dwarf mice. Aging Cell.

[15] Li F., Li Y., Liu H., Zhang X., Liu C., Tian K., Bolund L., Dou H., Yang W., Yang H. (2015). Transgenic Wuzhishan minipigs designed to express a dominant-negative porcine growth hormone receptor display small stature and a perturbed insulin/IGF-1 pathway. Transgenic Res..

[16] Yang H., Jiang Q., Wu D., Lan G., Fan J., Guo Y., Chen B., Yang X., Jiang H. (2015). Correlation analysis between expression levels of hepatic growth hormone receptor, janus kinase 2, insulin-like growth factor-i genes and dwarfism phenotype in Bama minipig. J. Nanosci. Nanotechnol..

[17] Boegheim I., Leegwater P., van Lith H.A., Back W. (2017). Current insights into the molecular genetic basis of dwarfism in livestock. Vet. J..

[18] Agarwal S.K., Cogburn L.A., Burnside J. (1994). Dysfunctional growth hormone receptor in a strain of sex-linked dwarf chicken: Evidence for a mutation in the intracellular domain. J. Endocrinol..

[19] Hull K.L., Harvey S. (1999). Growth hormone resistance: Clinical states and animal models. J. Endocrinol..

[20] Luo W., Lin S., Li G., Nie Q., Zhang X. (2016). Integrative analyses of miRNA-mRNA interactions reveal let-7b, miR-128 and MAPK pathway involvement in muscle mass loss in sex-linked dwarf chickens. Int. J. Mol. Sci..

[21] Fassone L., Corneli G., Bellone S., Camacho-Hubner C., Aimaretti G., Cappa M., Ubertini G., Bona G. (2007). Growth hormone receptor gene mutations in two Italian patients with Laron Syndrome. J. Endocrinol. Investig..

[22] Gennero I., Edouard T., Rashad M., Bieth E., Conte-Aurio F., Marin F., Tauber M., Salles J.P., El K.M. (2007). Identification of a novel mutation in the human growth hormone receptor gene (GHR) in a patient with Laron syndrome. J. Pediatr. Endocrinol. Metab..

[23] Derr M.A., Aisenberg J., Fang P., Tenenbaum-Rakover Y., Rosenfeld R.G., Hwa V. (2011). The growth hormone receptor (GHR) c.899dupC mutation functions as a dominant negative: Insights into the pathophysiology of intracellular GHR defects. J. Clin. Endocrinol. Metab..

[24] Waters M.J., Brooks A.J. (2011). Growth hormone receptor: Structure function relationships. Horm. Res. Paediatr..

[25] Yang N., Langenheim J.F., Wang X., Jiang J., Chen W.Y., Frank S.J. (2008). Activation of growth hormone receptors by growth hormone and growth hormone antagonist dimers: Insights into receptor triggering. Mol. Endocrinol..

[26] Baudet M.L., Harvey S. (2007). Small chicken growth hormone (scGH) variant in the neural retina. J. Mol. Neurosci..

[27] Barclay J.L., Kerr L.M., Arthur L., Rowland J.E., Nelson C.N., Ishikawa M., D’Aniello E.M., White M., Noakes P.G., Waters M.J. (2010). In vivo targeting of the growth hormone receptor (GHR) Box1 sequence demonstrates that the GHR does not signal exclusively through JAK2. Mol. Endocrinol..

[28] Wells J.A. (1996). Binding in the growth hormone receptor complex. Proc. Natl. Acad. Sci. USA.

[29] Behncken S.N., Waters M.J. (1999). Molecular recognition events involved in the activation of the growth hormone receptor by growth hormone. J. Mol. Recognit..

[30] De Vos A.M., Ultsch M., Kossiakoff A.A. (1992). Human growth hormone and extracellular domain of its receptor: Crystal structure of the complex. Science.

[31] Rosenbloom A.L. (2000). Physiology and disorders of the growth hormone receptor (GHR) and GH-GHR signal transduction. Endocrine.

[32] Liu Y., Jiang J., Lepik B., Zhang Y., Zinn K.R., Frank S.J. (2017). Subdomain 2, not the transmembrane domain, determines the dimerization partner of growth hormone receptor and prolactin receptor. Endocrinology.

[33] Huo S., Massova I., Kollman P.A. (2002). Computational alanine scanning of the 1:1 human growth hormone-receptor complex. J. Comput. Chem..

[34] Gent J., van Kerkhof P., Roza M., Bu G., Strous G.J. (2002). Ligand-independent growth hormone receptor dimerization occurs in the endoplasmic reticulum and is required for ubiquitin system-dependent endocytosis. Proc. Natl. Acad. Sci. USA.

[35] Brooks A.J., Dai W., O’Mara M.L., Abankwa D., Chhabra Y., Pelekanos R.A., Gardon O., Tunny K.A., Blucher K.M., Morton C.J. (2014). Mechanism of activation of protein kinase JAK2 by the growth hormone receptor. Science.

[36] Smit L.S., Meyer D.J., Billestrup N., Norstedt G., Schwartz J., Carter-Su C. (1996). The role of the growth hormone (GH) receptor and JAK1 and JAK2 kinases in the activation of Stats 1, 3, and 5 by GH. Mol. Endocrinol..

[37] Arman A., Yuksel B., Coker A., Sarioz O., Temiz F., Topaloglu A.K. (2010). Novel growth hormone receptor gene mutation in a patient with Laron syndrome. J. Pediatr. Endocrinol. Metab..

[38] Goncalves F.T., Fridman C., Pinto E.M., Guevara-Aguirre J., Shevah O., Rosembloom A.L., Hwa V., Cassorla F., Rosenfeld R.G., Lins T.S. (2014). The E180splice mutation in the GHR gene causing Laron syndrome: Witness of a Sephardic Jewish exodus from the Iberian Peninsula to the new world?. Am. J. Med. Genet. Part A.

[39] Ostrer H. (2016). The origin of the p.E180 growth hormone receptor gene mutation. Growth Horm. IGF Res..

[40] Cui D., Li F., Li Q., Li J., Zhao Y., Hu X., Zhang R., Li N. (2015). Generation of a miniature pig disease model for human Laron syndrome. Sci. Rep..

[41] Sobrier M.L., Dastot F., Duquesnoy P., Kandemir N., Yordam N., Goossens M., Amselem S. (1997). Nine novel growth hormone receptor gene mutations in patients with Laron syndrome. J. Clin. Endocrinol. Metab..

[42] Chen X., Song F., Dai Y., Bao X., Jin Y. (2003). A novel mutation of the growth hormone receptor gene (GHR) in a Chinese girl with Laron syndrome. J. Pediatr. Endocrinol. Metab..

[43] Putzolu M., Meloni A., Loche S., Pischedda C., Cao A., Moi P. (1997). A homozygous nonsense mutation of the human growth hormone receptor gene in a Sardinian boy with Laron-type dwarfism. J. Endocrinol. Investig..

[44] Fang P., Riedl S., Amselem S., Pratt K.L., Little B.M., Haeusler G., Hwa V., Frisch H., Rosenfeld R.G. (2007). Primary growth hormone (GH) insensitivity and insulin-like growth factor deficiency caused by novel compound heterozygous mutations of the GH receptor gene: Genetic and functional studies of simple and compound heterozygous states. J. Clin. Endocrinol. Metab..

[45] Moia S., Tessaris D., Einaudi S., de Sanctis L., Bona G., Bellone S., Prodam F. (2017). Compound heterozygosity for two GHR missense mutations in a patient affected by Laron Syndrome: A case report. Ital. J. Pediatr..

[46] Bonioli E., Taro M., Rosa C.L., Citana A., Bertorelli R., Morcaldi G., Gastaldi R., Coviello D.A. (2005). Heterozygous mutations of growth hormone receptor gene in children with idiopathic short stature. Growth Horm. IGF Res..

[47] Duriez B., Sobrier M.L., Duquesnoy P., Tixier-Boichard M., Decuypere E., Coquerelle G., Zeman M., Goossens M., Amselem S. (1993). A naturally occurring growth hormone receptor mutation: In vivo and in vitro evidence for the functional importance of the WS motif common to all members of the cytokine receptor superfamily. Mol. Endocrinol..

[48] Meyer S., Ipek M., Keth A., Minnemann T., von Mach M.A., Weise A., Ittner J.R., Nawroth P.P., Plöckinger U., Stalla G.K. (2007). Short stature and decreased insulin-like growth factor I (IGF-I)/growth hormone (GH)-ratio in an adult GH-deficient patient pointing to additional partial GH insensitivity due to a R179C mutation of the growth hormone receptor. Growth Horm. IGF Res..

[49] Kang J.H., Kim O.S., Kim J.H., Lee S.K., Park Y.J., Baik H.W. (2012). A novel mutation of exon 7 in growth hormone receptor mRNA in a patient with growth hormone insensitivity syndrome and neurofibromatosis type I. Int. J. Mol. Med..

[50] Diniz E.T., Jorge A.A., Arnhold I.J., Rosenbloom A.L., Bandeira F. (2008). Novel nonsense mutation (p.Y113X) in the human growth hormone receptor gene in a Brazilian patient with Laron syndrome. Arq. Bras. Endocrinol. Metabol..

[51] De Oliveira M.E., Lima C.H., Ogino L.L., Kasuki L., Gadelha M.R. (2016). Growth hormone receptor exon 3 isoforms may have no importance in the clinical setting of multiethnic Brazilian acromegaly patients. Pituitary.

[52] Rosenbloom A.L., Guevara-Aguirre J. (1998). Lessons from the genetics of laron syndrome. Trends Endocrinol. Metab..

[53] El K.M., Mella P., Rashad M., Buzi F., Meazza C., Zahra S., Elsedfy H.H. (2011). Growth hormone/IGF-I axis and growth hormone receptor mutations in idiopathic short stature. Horm. Res. Paediatr..

[54] Ying Y.Q., Wei H., Cao L.Z., Lu J.J., Luo X.P. (2007). Clinical features and growth hormone receptor gene mutations of patients with Laron syndrome from a Chinese family. Zhongguo Dang Dai Er Ke Za Zhi.

[55] Tiulpakov A., Rubtsov P., Dedov I., Peterkova V., Bezlepkina O., Chrousos G.P., Hochberg Z. (2005). A novel C-terminal growth hormone receptor (GHR) mutation results in impaired GHR-STAT5 but normal STAT-3 signaling. J. Clin. Endocrinol. Metab..

[56] Meacham L.R., Brown M.R., Murphy T.L., Keret R., Silbergeld A., Laron Z., Parks J.S. (1993). Characterization of a noncontiguous gene deletion of the growth hormone receptor in Laron’s syndrome. J. Clin. Endocrinol. Metab..

[57] Yang C., Chen J.Y., Lai C.C., Lin H.C., Yeh G.C., Hsu H.H. (2004). Clinical, biochemical and molecular investigations of three Taiwanese children with Laron syndrome. J. Pediatr. Endocrinol. Metab..

[58] Wojcik J., Berg M.A., Esposito N., Geffner M.E., Sakati N., Reiter E.O., Dower S., Francke U., Postel-Vinay M.C., Finidori J. (1998). Four contiguous amino acid substitutions, identified in patients with Laron syndrome, differently affect the binding affinity and intracellular trafficking of the growth hormone receptor. J. Clin. Endocrinol. Metab..

[59] Hattori A., Katoh-Fukui Y., Nakamura A., Matsubara K., Kamimaki T., Tanaka H., Dateki S., Adachi M., Muroya K., Yoshida S. (2017). Next generation sequencing-based mutation screening of 86 patients with idiopathic short stature. Endocr. J..

[60] Jorge A.A., Souza S.C., Arnhold I.J., Mendonca B.B. (2004). The first homozygous mutation (S226I) in the highly-conserved WSXWS-like motif of the GH receptor causing Laron syndrome: Supression of GH secretion by GnRH analogue therapy not restored by dihydrotestosterone administration. Clin. Endocrinol..

[61] Hopp M., Rosenbloom A.L., Griffiths J., Kgwete S., Vaccarello M.A. (1996). Growth hormone receptor deficiency (Laron syndrome) in black African siblings. S. Afr. Med. J..

[62] Akinci A., Rosenfeld R.G., Hwa V. (2013). A novel exonic GHR splicing mutation (c.784G > C) in a patient with classical growth hormone insensitivity syndrome. Horm. Res. Paediatr..

[63] Vairamani K., Merjaneh L., Casano-Sancho P., Sanli M.E., David A., Metherell L.A., Savage M.O., Del P.J., Backeljauw P.F., Rosenfeld R.G. (2017). Novel dominant-negative GH receptor mutations expands the spectrum of GHI and IGF-I deficiency. J. Endocr. Soc..

[64] Billestrup N., Bouchelouche P., Allevato G., Ilondo M., Nielsen J.H. (1995). Growth hormone receptor C-terminal domains required for growth hormone-induced intracellular free Ca^2+^ oscillations and gene transcription. Proc. Natl. Acad. Sci. USA.

[65] Klammt J., Shen S., Kiess W., Kratzsch J., Stobbe H., Vogel M., Luo F., Pfaffle R. (2015). Clinical and biochemical consequences of an intragenic growth hormone receptor (GHR) deletion in a large Chinese pedigree. Clin. Endocrinol..

[66] Feigerlova E., Swinyard M., Derr M.A., Farnsworth J., Andrew S.F., Rosenfeld R.G., Hwa V. (2013). A novel GHR intronic variant, c.266+83G>T, activates a cryptic 5’ splice site causing severe GHR deficiency and classical GH insensitivity syndrome. Horm. Res. Paediatr..

[67] Iida K., Takahashi Y., Kaji H., Nose O., Okimura Y., Abe H., Chihara K. (1998). Growth hormone (GH) insensitivity syndrome with high serum GH-binding protein levels caused by a heterozygous splice site mutation of the GH receptor gene producing a lack of intracellular domain. J. Clin. Endocrinol. Metab..

[68] Moffat J.G., Edens A., Talamantes F. (1999). Structure and expression of the mouse growth hormone receptor/growth hormone binding protein gene. J. Mol. Endocrinol..

[69] Elzein S., Goodyer C.G. (2014). Regulation of human growth hormone receptor expression by microRNAs. Mol. Endocrinol..

[70] Dastot F., Sobrier M.L., Duquesnoy P., Duriez B., Goossens M., Amselem S. (1996). Alternatively spliced forms in the cytoplasmic domain of the human growth hormone (GH) receptor regulate its ability to generate a soluble GH-binding protein. Proc. Natl. Acad. Sci. USA.

[71] Amselem S., Duquesnoy P., Duriez B., Dastot F., Sobrier M.L., Valleix S., Goossens M. (1993). Spectrum of growth hormone receptor mutations and associated haplotypes in Laron syndrome. Hum. Mol. Genet..

[72] Chatterjee S., Shapiro L., Rose S.J., Mushtaq T., Clayton P.E., Ten S., Bhangoo A., Kumbattae U., Dias R., Savage M.O. (2018). Phenotypic spectrum and responses to recombinant human IGF1 (rhIGF1) therapy in patients with homozygous intronic pseudoexon growth hormone receptor mutation. Eur. J. Endocrinol..

[73] Singhal V., Goh B.C., Bouxsein M.L., Faugere M.C., DiGirolamo D.J. (2013). Osteoblast-restricted disruption of the growth hormone receptor in mice results in sexually dimorphic skeletal phenotypes. Bone Res..

[74] Yakar S., Isaksson O. (2016). Regulation of skeletal growth and mineral acquisition by the GH/IGF-1 axis: Lessons from mouse models. Growth Horm IGF Res..

[75] Liu Z., Mohan S., Yakar S. (2016). Does the GH/IGF-1 axis contribute to skeletal sexual dimorphism? Evidence from mouse studies. Growth Horm. IGF Res..

[76] Wit J.M., Camacho-Hubner C. (2011). Endocrine regulation of longitudinal bone growth. Endocr. Dev..

[77] Nielsen R.H., Clausen N.M., Schjerling P., Larsen J.O., Martinussen T., List E.O., Kopchick J.J., Kjaer M., Heinemeier K.M. (2014). Chronic alterations in growth hormone/insulin-like growth factor-I signaling lead to changes in mouse tendon structure. Matrix Biol..

[78] Gevers E.F., van der Eerden B.C., Karperien M., Raap A.K., Robinson I.C., Wit J.M. (2002). Localization and regulation of the growth hormone receptor and growth hormone-binding protein in the rat growth plate. J. Bone Miner. Res..

[79] Mavalli M.D., DiGirolamo D.J., Fan Y., Riddle R.C., Campbell K.S., van Groen T., Frank S.J., Sperling M.A., Esser K.A., Bamman M.M. (2010). Distinct growth hormone receptor signaling modes regulate skeletal muscle development and insulin sensitivity in mice. J. Clin. Investig..

[80] Vijayakumar A., Buffin N.J., Gallagher E.J., Blank J., Wu Y., Yakar S., LeRoith D. (2013). Deletion of growth hormone receptors in postnatal skeletal muscle of male mice does not alter muscle mass and response to pathological injury. Endocrinology.

[81] Lucy M.C. (2012). Growth hormone regulation of follicular growth. Reprod. Fertil. Dev..

[82] Ranke M.B., Wit J.M. (2018). Growth hormone—Past, present and future. Nat. Rev. Endocrinol..

[83] Yakar S., Werner H., Rosen C. (2018). Insulin-like growth factors: Actions on the skeleton. J. Mol. Endocrinol..

[84] Lee S.S., Han A., Ahn M.B., Kim S.H., Cho W.K., Cho K.S., Park S.H., Jung M.H., Suh B. (2017). Growth without growth hormone in combined pituitary hormone deficiency caused by pituitary stalk interruption syndrome. Ann. Pediatr. Endocrinol. Metab..

[85] Walters T.D., Griffiths A.M. (2009). Mechanisms of growth impairment in pediatric Crohn’s disease. Nat. Rev. Gastroenterol. Hepatol..

[86] Johnston L.B., Pashankar F., Camacho-Hubner C., Savage M.O., Clark A.J. (2000). Analysis of the intracellular signalling domain of the human growth hormone receptor in children with idiopathic short stature. Clin. Endocrinol..

[87] Hujeirat Y., Hess O., Shalev S., Tenenbaum-Rakover Y. (2006). Growth hormone receptor sequence changes do not play a role in determining height in children with idiopathic short stature. Horm. Res..

[88] Kijas J.M., Wales R., Tornsten A., Chardon P., Moller M., Andersson L. (1998). Melanocortin receptor 1 (MC1R) mutations and coat color in pigs. Genetics.

[89] Lau J.S., Yip C.W., Law K.M., Leung F.C. (2007). Cloning and characterization of chicken growth hormone binding protein (cGHBP). Domest. Anim. Endocrinol..

[90] Agarwal S.K., Cogburn L.A., Burnside J. (1995). Comparison of gene expression in normal and growth hormone receptor-deficient dwarf chickens reveals a novel growth hormone regulated gene. Biochem. Biophys. Res. Commun..

[91] Zhang L., Lin S., An L., Ma J., Qiu F., Jia R., Nie Q., Zhang D., Luo Q., Li T. (2016). Chicken GHR natural antisense transcript regulates GHR mRNA in LMH cells. Oncotarget.

